# Pharmacological Mechanisms of Shangke Huangshui against Skin and Soft Tissue Infection

**DOI:** 10.1155/2022/9312611

**Published:** 2022-02-16

**Authors:** Yanfen Chen, Mengqiu Li, Fanghao Zheng, Wei Jiang, Kaijun Lei, Huaiguo Li, Dongwen Liu, Bairong Zhang, Mingfeng He

**Affiliations:** ^1^School of Traditional Chinese Medicine, Guangdong Pharmaceutical University, Guangzhou 510006, Guangdong, China; ^2^Foshan Hospital of Traditional Chinese Medicine, Foshan 528000, Guangdong, China

## Abstract

**Background:**

Skin and soft tissue infections (SSTIs) are a group of common diseases, usually caused by bacteria. Shangke Huangshui (SK) has been widely used to treat various SSTIs diseases for many years, but its mechanism is unclear. Therefore, this study was designed to investigate the anti-infective effect of SK on different skin and soft tissue infection diseases and to explore its underlying mechanism.

**Methods:**

The subcutaneous abscess mouse model, the dermal ulcer rat model, and the infectious soft tissue injury rat model were established by injection of *Staphylococcus aureus* to evaluate the anti-inflammatory and antibacterial effects of SK. Abscess volume, local appearance score and histological changes, bacterial contents, and hydroxyproline concentration in the skin or soft tissue were analyzed. The levels of NO, TNF-*α*, IL-1*β*, and IL-8 in the serum and tissue were determined by ELISA method. The mRNA expression levels of TLR2, MyD88, TAK1, NF-*κ*B, AP-1, and other genes were measured with qRT-PCR method, and the protein expression of TLR2, MyD88, TAK1, NF-*κ*B, and AP-1 was detected by western blot method.

**Results:**

SK had an obvious therapeutic effect on skin and soft tissue infections. It reduced the volume of abscess and promoted the healing of skin ulcer, improved pathological phenomena, such as inflammatory infiltration of skin and soft tissue, reduced the levels of white blood cells and NO in the blood, decreased bacteria contents in the skin and soft tissue. Furthermore, SK decreased the mRNA expression of TLR2, MyD88, TAK1, NF-*κ*B and AP-1, and other related genes and also downregulated the protein expression of TLR2, MyD88, TAK1, NF-*κ*B, and AP-1.

**Conclusion:**

The experiments provide evidence that SK can treat skin and soft tissue infection diseases based on its comprehensive antibacterial and anti-inflammatory effects. The therapeutic mechanism may be associated with the inhibition of TLR2/MyD88/NF-*κ*B signaling pathway.

## 1. Introduction

Skin and soft tissue infections (SSTIs) are a group of common inflammatory disease. Usually, the epidermis, dermis, and subcutaneous tissue are infected with pathogens of purulence. These diseases are clinical entities of variable presentations, etiologies, and severities, which involve microbial invasion of the skin and underlying soft tissues, ranging from mild infections, such as impetigo or ecthyma, to life-threatening infections, such as necrotizing fasciitis. Therefore, they account for substantial morbidity, and a large percentage of infections require hospitalization [1, 2]. It is accepted that *Staphylococcus aureus* (*S. aureus*), a Gram-positive bacterial pathogen, is one of the most important pathogens in SSTIs, which most commonly affects the lower limbs, perineum, groin, buttocks, hands, or feet, ranging from mild diseases to life-threatening conditions [3, 4]. And the increase in SSTIs because of multidrug-resistant pathogens has become a global concern [5, 6]. Therefore, an efficacious medicine to treat SSTIs in humans is needed, but the problem of frequent overtreatment with the use of broad-spectrum antibiotics has proven to be increasingly pronounced [7]. Nevertheless, the application of antibiotic was limited by the adverse drug reactions of antibiotics and the increasingly bacterial resistance. Herein, some traditional treatments have played a role in treating SSTIs, such as traditional Chinese medicine (TCM). TCM displays multiple target sites and little toxic effects and has been confirmed to be effective in the treatment of infections caused by certain bacteria, such as salmonella [8, 9] and *Escherichia coli* [10, 11]. Chinese medicine compound preparations have increasingly become a research hotspot with varieties of biologically active ingredients, multiefficacy in clinical application and broad antibacterial spectrum.

Shangke Huangshui (SK), also known as yellow herbal lotion, is a traditional Chinese herbal prescription used in hospitals for more than 60 years. The recipe is mainly composed of herbs such as coptidis ardeni (Huanglian, Co*ptis chinensis* Franch.), phellodendri chinensis cortex (Huangbai, *Phellodendron chinense* Schneid.), ardenia fructus (Zhi-zi, *Ganiema jasminoides* Ellis), and arnebiae radix (Zicao, *Arnebia euchroma* (Royle) Johnst.) [12]. It has been proved that SK could clear heat and remove toxicity, promote blood circulation to relieve pain, relieve swelling and promote granulation, and has been widely used to treat closed or open trauma, wound infection in clinical practice [13, 14]. Studies reported that SK can not only reduce the inflammatory marks, VAS score, and clinical symptom score of patients with acute gouty arthritis [15] but also be used effectively in the treatment of infectious bone defects after tibial trauma for it can eliminate swellings and pains of limbs, shorten the time of preoperative detumescence, and reduce postoperative pin-related infection [16]. Moreover, the external application of SK had more control over the infection, reduced inflammation and promoted wound healing compared with Anerdian antibacterial lotion (III), and showed a good efficacy in the treatment of lower extremity posttraumatic wound infection [17].

Although the clinical application of SK is very extensive and the curative effect is good, its related mechanism is still unclear. Our previous studies have shown that SK has anti-inflammatory and anti-acute soft tissue injury effects. In this study we evaluated the curative efficacy and its underlying mechanism in various models of SSTIs, including subcutaneous abscess, dermal ulcer, and surgical soft tissue infection.

## 2. Materials and Methods

### 2.1. Preparation of SK

This compound preparation is an herbal mixture of coptidis rhizoma (Huanglian, Coptis chinensis Franch.), phellodendri chinensis cortex (Huangbai, Phellodendron chinense Schneid.), gardeniae fructus (Zhi-zi, Ganiema jasminoides Ellis), arnebiae radix (Zicao, *Arnebia euchroma* (Royle) Johnst.), and so on. All herbs were qualified according to the standards noted in “The Pharmacopoeia of the People's Republic of China, 2015.” The preparation was prepared by the preparation center of Foshan hospital of traditional Chinese medicine, with the quality controlled as the references previously described [18].

### 2.2. Preparation of Infectious Bacterial Solution

The *S. aureus* strain (ATCC6538) was obtained from laboratory of microbiology and immunology in basic college of Guangdong Pharmaceutical University, incubated in 37°C constant temperature incubator and nutrient agar medium for 24 h. The bacterial fluid was harvested and counted by UV spectrophotometer, then diluted with sterile normal saline to the concentrations of 1 × 10^14^ CFU·L^−1^, and stored at 4°C before use.

### 2.3. Experimental Animals

Male Kunming mice (four weeks old, 18–22 g) and male Sprague Dawley rats (six weeks old, 180–220 g) were purchased separately from the Medical Experimental Animal Center of Guangzhou University of Chinese Medicine (license no. SCXK 2013–0034, Guangdong, China) and Laboratory Animal Center of Southern Medical University (license no. SCXK 2016–0041, Guangdong, China). All of the mice and rats were maintained in 12 h light-dark cycle at controlled temperature (22–23°C) and relative humidity (50–60%) in the animal experiment center of Guangdong Pharmaceutical University. Food and water were provided ad libitum. All procedures involving animals were in accordance with the Regulations of Experimental Animals Administration issued by the Ministry of Science and Technology of People's Republic of China, and the use of laboratory animals was approved by the Ethics Committee of Experimental Animals of Guangdong Pharmaceutical University (gdpulacspf2017136).

### 2.4. Mice Model of Subcutaneous Abscess Formation

The animals were anesthetized with 0.5% pentobarbital sodium, and their backs were shaved and disinfected with betadine and 70% ethanol. According to [19], each mouse destined to be assigned to a subcutaneous abscess formation group received a subcutaneous injection of 0.1 ml infectious bacteria solution (5 × 10^12^ CFU·L^−1^) prepared as previously described. Subcutaneous abscess was evaluated 48 hours after inoculation of *S. aureus* solution to determine whether it was modeled. Modeling animals were randomly assigned into five equal groups (12 mice in each group): the model group (Mod), positive group (Mupirocin ointment, Mup), and three SK groups (high, medium, and low concentration group). Then, in the infection site, the model group had a gauze with normal saline applied, the positive medicine group had a gauze with 0.1 g mupirocin applied, and the SK-H group, the SK-M group, and the SK-L group had a gauze with 0.2 ml of SK (1.3 g/ml, 0.65 g/ml, 0.13 g/ml) applied twice a day separately. During the experiment, the changes of body weight and skin appearance of mice were observed and recorded every day. The long diameter and wide diameter of back abscess of each mouse were measured with vernier caliper, and the abscess volume was calculated according to the formula: *V*=*π*/6 × *L* × *W*^2^ (*V* is the abscess volume, *L* is the long diameter of abscess, and W is the wide diameter of abscess).

### 2.5. Rats Model of Dermal Ulcer

Rats were anesthetized, shaved, and disinfected as described previously. A circular incision (1.5 cm in diameter) was made on the back skin of each rat, reaching the fascia layer, and then inoculated with 0.2 ml of *S. aureus* solution (1 × 10^13^ CFU·L^−1^). 24 h later, the modeling rats were randomly divided into five groups: the model group (Mod), positive group (Mupirocin ointment, Mup, 0.2 g), and three SK groups. The SK‐H group, SK‐M group, and SK‐L group were given gauze containing 1mL of (1.3 g/ml, 0.65 g/ml, and 0.13 g/ml). Each group received the previously mentioned treatment and administration, respectively. In this test, the changes of body weight and skin appearance of rats were observed and recorded every day. The skin appearance was scored according to the scoring criteria recorded in the literature [20]. The main observation points were as follows: (1) swelling and/or erythema, (2) purulent secretion, and (3) the status of nascent granulation tissue. Each evaluation point was divided into four levels according to the severity: 0 was normal, 1 was mild, 2 was moderate, and 3 was severe.

### 2.6. Rats Model of Infectious Soft Tissue Injury

Rats were anesthetized, shaved, and disinfected as previously described. The thigh skin and gastrocnemius muscle of rats were aseptically cut with a scalpel, and each wound was injected with 0.05 ml *S. aureus* bacterial solution (2 × 10^9^ CFU·L^−1^) and then sutured. 24 h later, the animals were randomly assigned into five equal groups (12 rats in each group): the model group (Mod), positive group (Yunnan Baiyao, YN, 1 ml), and three SK groups (1.3 g/ml, 0.65 g/ml, and 0.13 g/ml). Each group received the previously mentioned treatment and administration, respectively. The changes in body weight and local soft tissue appearance of rats were observed and recorded every day. The soft tissue appearance was scored according to the scoring criteria [21]. The main observation points were as follows: (1) subcutaneous ecchymosis, (2) skin and muscle color, (3) skin and muscle swelling, and (4) movement disorder. Each evaluation content was divided into four levels according to the severity: 0 was normal, 1-2 was mild, 3 was moderate, and 4 was severe. Then, the scores were summed to obtain a composite score for each animal, which was defined as the severity of infectious soft tissue injury.

### 2.7. Histological Examination

The skin and muscle adipose tissues were fixed in 4% paraformaldehyde for 24 h and processed for paraffin sections. Tissue sections (5 *µ*m) were cut and stained with hematoxylin and eosin for examination by microscopy.

### 2.8. Etiological Examination of Skin and Soft Tissue

It was determined by dilution method of plate counting. Briefly, the infected skin or muscle tissue was excised, weighed, homogenized, and cultured quantitatively. Serial dilutions of the homogenates (at the dilution rate from 10^1^ to 10^6^ times) were cultured and inoculated in nutrient agar culture medium at 37°C for 24 h. Then, the contents of *S. aureus* were counted, respectively.

### 2.9. Detection of Blood and Tissue Related Indexes

During the experiment, blood was collected from the orbit of rats, and the level of blood leukocytes (White blood cell, WBC) was detected by counting method. After the experiment, rats were anesthetized and blood was obtained from the abdominal aorta. The blood was allowed to clot and the serum separated. Lysozyme and NO level in blood was measured in accordance with methods provided by the kits (Nanjing Jiancheng Bioengineering Institute). The levels of hydroxyproline (HYP), IL-8, TNF-*α*, and IL-1*β* in the infected skin or muscle tissues were also detect by the kits (Nanjing Jiancheng Bioengineering Institute).

### 2.10. Quantitative Reverse Transcription PCR (qRT-PCR) Analysis

Total RNA of each animal was extracted from the infected skin or the muscle tissue. After the amount of total RNA was measured, reverse-transcription of total RNA was performed using the GoScript™ RT system (Promega, USA). Relative target gene transcription was performed using SYBR Green qpcr Mix (DSBIO, China) and quantitative real-time PCR (CFX96 Connect Real-Time System, Bio-Rad, USA). The PCR specific primers for TLR2, MyD88, IRAK1, TRAF6, TAK1, p38, JNK, NF-*κ*B, AP-1, IL8, and GAPDH were shown as follows. PCR was performed using the following conditions: denaturing at 94°C for 3 min, followed by 40 cycles of 94°C for 15 s, 60°C for 15 s, and 72°C for 20 s.

The transcription of GAPDH was measured in parallel with each sample as housekeeping gene, and the blank control was tested for each gene. The relative level of each gene was calculated and presented as the ratio of GAPDH. Primers for related gene were as follows: GAPDH, F: 5′-AACGACCCCTTCATTGACCTC-3′, R: 5′-CCTTGACTGTGCCGTTGAACT-3′; TLR2, F: 5′-TGTCATGTGATGCTGCTG GTGTG-3′, R: 5′-ATTGTGTTGATTCCGCTGGACTCC-3′; MyD88, F: 5′-TGGCGGAGGAGATGGGTTTCG-3′, R: 5′-AGCCTGCCGACCGACGAG-3′; IRAK1, F: 5′-GTCCTCTGCCTCCACCTTCCTC-3′, R: 5′-GCTCTCTGGGCTTGGCTTGATG-3′; TRAF6, F: 5′-GAATCACTTGGCACGGCACTTG-3′, R: 5′-TGGAGAGGAGGCATCGCATGG-3′; TAK1, F: 5′-CTCGTCCTCCTCCTCGTCTTCTG-3′, R: 5′-ACCTCTTCCACCTCGATCTCC TTG-3′; AP-1, F: 5′-CAGCCGCCGCACCACTTG-3′, R: 5′-TCCGCTCCTGAGACTCCATGT C-3′; JNK, F: 5′-TCTCAGCATCCGGTCTCTTCGC-3′, R: 5′-CTACAGCAGCCCAGAGGT CCAG-3′; P38, F: 5′-TGCGGCTGCTGAAGCACATG-3′, R: 5′-AACTGAACG TGGTCATCGGTAAGC-3′; NF-*κ*B, F: 5′-GCTGCCAACATCATCCAGGAAGG-3′, R: 5′-TGATGCCAGAGCGGCTACTCAG-3′; IL-8, F: 5′-CGTCTTGGCAGCAGTCCTTCTC-3′, R: 5′-TCTGAATTGGCACAGCGTGGTC-3′; TNF-*α*, F: 5′-GCAGGACTTCTTCAGCGG ACATG-3′, R: 5′-GTTAGGTTCAGCTCGCCTCTTCAC-3′.

### 2.11. Western Blotting

Muscle tissues were homogenized in RIPA cell lysis buffer to extract the total protein. Protein concentration was determined using BCA Protein Assay Kit (Beyotime, China). Aliquots of each protein sample (30 *μ*g) were used to measure the expression levels of TLR2, MyD88, TAK1, NF-*κ*B, AP1, and *β*-actin by separating in 10% SDS-polyacrylamide gel electrophoresis. The proteins were separated by SDS-PAGE and transferred onto a polyvinylidene difluoride (PVDF) membrane. The membrane was incubated for 2 h with 5% skim milk at room temperature and then probed overnight at 4°C with the specific primary antibodies, followed by incubating with the appropriate second antibodies (1:5000) at room temperature for 2 h. After washing with TBST three times, the blots were exposed to ECL reagent. After washing with TBST three times, the blots were imaged by exposing to ECL reagent, and then, the results were normalized using the quantities of *β*-actin.

### 2.12. Statistical Analysis

All the results were expressed as the mean ± standard deviation. Statistical analyses were performed utilizing the SPSS™ ver. 24.0 statistical package, and statistical differences were determined by means of analysis of variance (ANOVA) and performed with control group. Significant differences are presented as ^*∗*^*P* < 0.05 and ^*∗∗*^*P* < 0.01.

## 3. Results

### 3.1. Effect of SK on Subcutaneous Abscess Mice Model

#### 3.1.1. Abscess Volume

**Figure 1:**
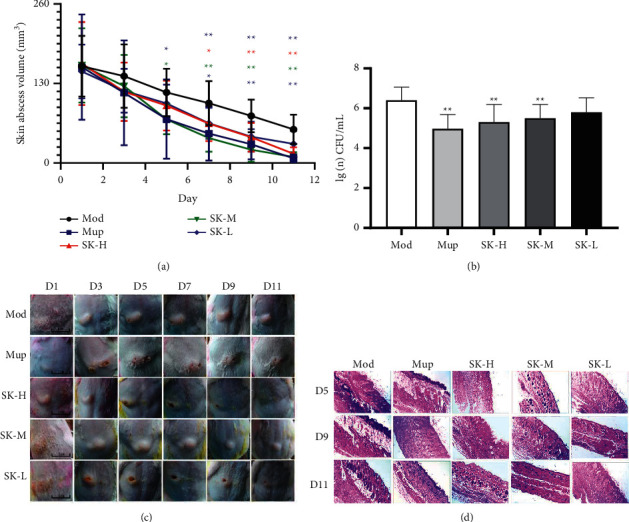
SK alleviated *S. aureus*-induced subcutaneous abscess in mice. (a) Abscess volume in mice. (b) The bacterial contents in the skin of mice. (c) Subcutaneous abscess appearances. (d) Hematoxylin and eosin staining of skin tissues (40×). Values are expressed as mean ± SD in each group. ^*∗*^*P* < 0.05 and ^*∗∗*^*P* < 0.01 versus Mod group. (*n* = 10).

The abscess appeared on the back of the mice after *S. aureus* solution inoculating, and some of them were ruptured and flowed out in 2–4 days. Compared with the model group, the abscess volume of the SK-M group and Mup group both reduced obviously after treating for five days (*P* < 0.05); on days 9 and 11 after the treatment, the abscesses of the SK-H, SK-M, SK-L, and Mup groups were significantly smaller than the model group (*P* < 0.01), which indicated that SK had therapeutic effect on the skin abscess of mice (Figures 1(a) and 1(c)).

#### 3.1.2. Bacterial Contents in the Skin of Mice

After 11 days of administration, the skin of the abscess was taken for bacterial reverse culture. The experimental data showed that the bacterial content of SK-H, SK-M, and Mup group was significantly lower than the model group (*P* < 0.01), and the bacterial content in the SK-L group was also lower than that of model group but had no significant difference. This result indicated that SK and Mup could inhibit the growth of *S. aureus* (Figure 1(b)).

#### 3.1.3. Pathological Examination

On day five after the treatment, the skin infected at the abscess site was damaged to varying degrees, the cell structures of derma and subcutaneous tissue were not clear, the gap between skin tissue cells became larger, and obvious inflammatory cell infiltration in the skin tissues was observed. On day nine, compared with the model group, the number of inflammatory cells decreased significantly in the treatment groups, except for SK-L group, and the cell structures of derma and subcutaneous tissue were not clear yet. On day 11, mice in the treatment groups showed no abscess and slight inflammatory cell infiltration, and scar tissue could be seen; however, massive inflammatory cell infiltration and broken collagen fibers could be observed in the model group (Figure 1(d)). The results indicated that SK could accelerate the regression of skin abscess and reduce the infiltration of inflammatory cells.

### 3.2. Effect of SK on Dermal Ulcer Rat Model

#### 3.2.1. SK Alleviated the Symptoms and Signs of Dermal Ulcer Rat Model

**Figure 2:**
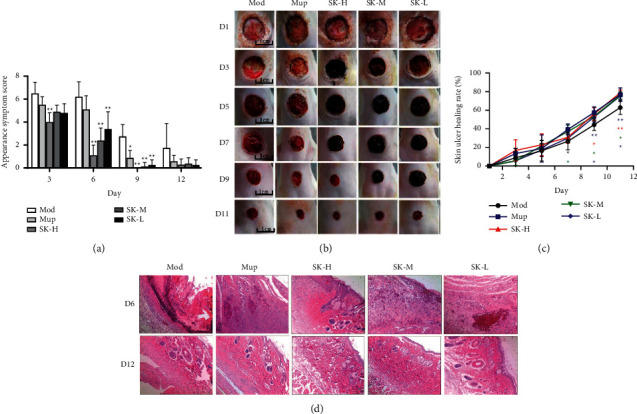
SK alleviated *S. aureus*-induced dermal ulcer in rats. (a) Clinical symptoms and signs were tested as dermal ulcer scores. (b) Redness, inflammation, purulent secretion, and other phenomena in the skin ulcer site. (c) The dermal ulcer healing rate of rats. (d) Hematoxylin and eosin staining of dermal ulcer tissues (40×). Values are expressed as mean ± SD in each group. ^*∗*^*P* < 0.05 and ^*∗∗*^*P* < 0.01 versus Mod group (*n* = 8).

In our study, *S. aureus* was inoculated in the skin wounds of rat back to assess ulcer symptom (Figure 2(b)). As shown in Figure 2(a), on the third day of administration, compared with the model group, the scores of redness, purulent secretion, and inflammatory phenomenon in the SK-H group were significantly lower than that in the Mod group (*P* < 0.01). On day six, the rats in the Mod group and YN group were partially scabbed, and the purulent secretion was reduced after scabbing, while the rats in the SK groups were basically scabbed, and the purulent secretion and inflammatory infiltration were less. On days six and nine, the symptoms of redness, inflammation, and purulent secretion in the SK groups were lighter, and the score of ulcer appearance symptoms was significantly lower (*P* < 0.01). On day 12, most of the scabs in the SK groups were removed, and the scabs were slightly red, basically without purulent secretion. There were more scabs in the Mup group, and a small part of the scabs in the model group was removed. Most of the scabs were growing, showing that the anti-inflammatory effect was obvious in the rats treated with SK.

#### 3.2.2. Effects of the Dermal Ulcer Healing Rate in Rats

On day two, scab appeared in the SK groups. On day 5, the dermal ulcer healing of SK groups began to accelerate. On day 7, compared with the model group, the dermal ulcer healing rate of SK-M group and Mup group had obvious difference (*P* < 0.05), and on days 9 and 11, the healing rate of SK groups and Mup group showed statistical difference compared with that of model group (*P* < 0.05 or *P* < 0.01). Moreover, the rate of SK-H group was higher than that of Mup group. It indicated that SK helps make the scabs generate and desquamate and then accelerate the dermal ulcer healing (Figure 2(c)).

#### 3.2.3. Pathological Examination

On day six, in the model group, the overall structures of skin were significantly changed and not complete, and a lot of inflammatory cells infiltrated, and broken collagen fibers could be observed in the skin. However, in the SK-M and SK-H groups, granulation tissue was evident, and the skin structure was relatively complete. In addition, the SK-M group showed slight inflammatory cell infiltration. On day 12, the skin structure of each group was relatively complete, but in the model group, the cell gap was larger, and the inflammatory infiltration was more obvious than that in the treatment groups (Figure 2(d)).

#### 3.2.4. Bacterial Contents in the Skin of Rats

**Figure 3:**
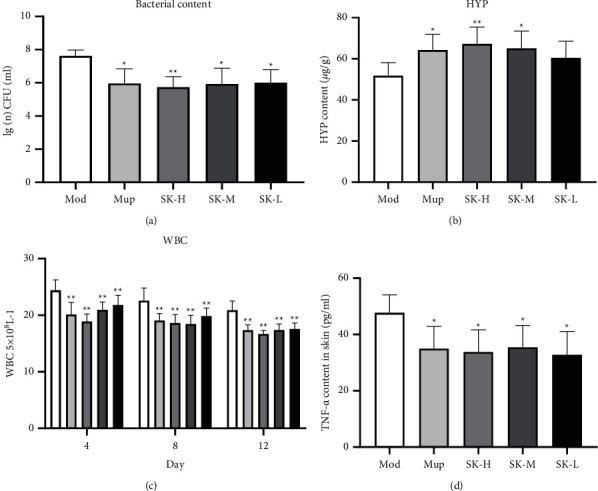
SK treatment ameliorated dermal ulcer in rats. (a) The bacterial contents of skin ulcer tissue on the 12^th^ day of administration. (b) Hydroxyproline contents of skin ulcer tissue on the 12^th^ day. (c) Leukocyte level of rats every four days. (d) TNF-*α* level in the skin ulcer tissue on the 12^th^ day. Values are expressed as mean ± SD in each group. ^*∗*^*P* < 0.05 and ^*∗∗*^*P* < 0.01 versus Mod group (*n* = 8).

The experimental data showed that the bacteria content in the skin of rats with SK-M, SK-L, or Mup treatment was significantly lower than that in model group (*P* < 0.05), and there were highly dominant differences of that among SK-H and model groups (*P* < 0.01), which indicated that SK and Mup can inhibit the growth of *S. aureus* (Figure 3(a)).

#### 3.2.5. Hydroxyproline Contents of the Skin

Hydroxyproline accounts for 13.4% of collagen and is the sensitive biochemical marker reacting collagen fibers changes. Most of the collagen is distributed in skin, tendon, cartilage, and blood vessel, and therefore, the content of hydroxyproline can reflect the collagen metabolism of connective tissue [22], and the high content of hydroxyproline can accelerate the healing of ulcer. On day 12 after the treatment, compared with the model group, the content of hydroxyproline in the SK-H group and SK-M group increased significantly (*P* < 0.01 or *P* < 0.05), indicating that the content of collagen in the ulcer site increased in the process of ulcer recovery, so as to accelerate the healing of skin ulcer (Figure 3(b)).

#### 3.2.6. Effect of SK on Leukocyte Level

During this experiment, the level of leukocytes in blood was the highest on day four, and it suggested that inflammation is more obvious in the early stage. On days 4, 8, and 12, the leukocyte levels of all treatment groups significantly decreased compared with the model group (*P* < 0.01), which indicated SK had a good effect on inflammation (Figure 3(c)).

#### 3.2.7. Effect of SK on TNF-*α* Level

Inflammatory cells release a large number of inflammatory mediators and cytokines, such as TNF-*α* and IL-1*β*, which are the early inflammatory factors that mediate the inflammatory response. The experiment showed that the level of TNF-*α* in the skin ulcer tissue of rats in the administration groups both decreased. Compared with the Mod group, the contents of TNF-*α* in the skin ulcer tissue of the rats with SK or Mup treatment were significantly lower (*P* < 0.05) (Figure 3(d)).

#### 3.2.8. Expression of TAK1, NF-*κ*B, p38, and TNF-*α* mRNA in Skin

TAK1, NF-*κ*B, p38, and TNF-*α* are important regulatory genes of inflammation, which play an important role in inflammation. Activation of TAK1 can promote the activation of p38 and NF-*κ*B pathway and promote the release of IL-12, IL-8 and TNF-*α*, to aggravate the inflammatory response.

**Figure 4:**
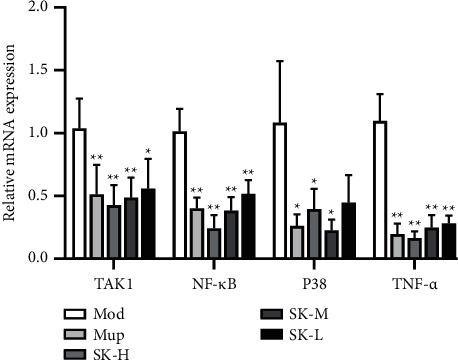
The expression of TAK1, NF-*κ*B P38, and TNF-*α* mRNA in skin ulcer tissues (qPCR). Values are expressed as mean ± SD in each group. ^*∗*^*P* < 0.05 and ^*∗∗*^*P* < 0.01 versus Mod group (*n* = 7).

The results demonstrated that the expression of NF-*κ*B and TNF-*α* in the treatment groups decreased markedly than that in the model group (*P* < 0.01). Furthermore, compared with the model group, the expression of TAK1 was significantly decreased in SK-H, SK-M or Mup groups (*P* < 0.01) and lowered in SK-L group (*P* < 0.05). The expression of p38 also decreased evidently in SK-H, SK-L, or Mup groups (*P* < 0.05; Figure 4). The previously mentioned results manifested that SK can block the activation of p38 and NF-*κ*B signaling pathway and inhibit the expression of genes upstream, such as TAK1.

### 3.3. Effect of SK on Soft Tissue Infection Rats Model

#### 3.3.1. SK Alleviated the Symptoms of Infectious Soft Tissue Injury Rat Model

**Figure 5:**
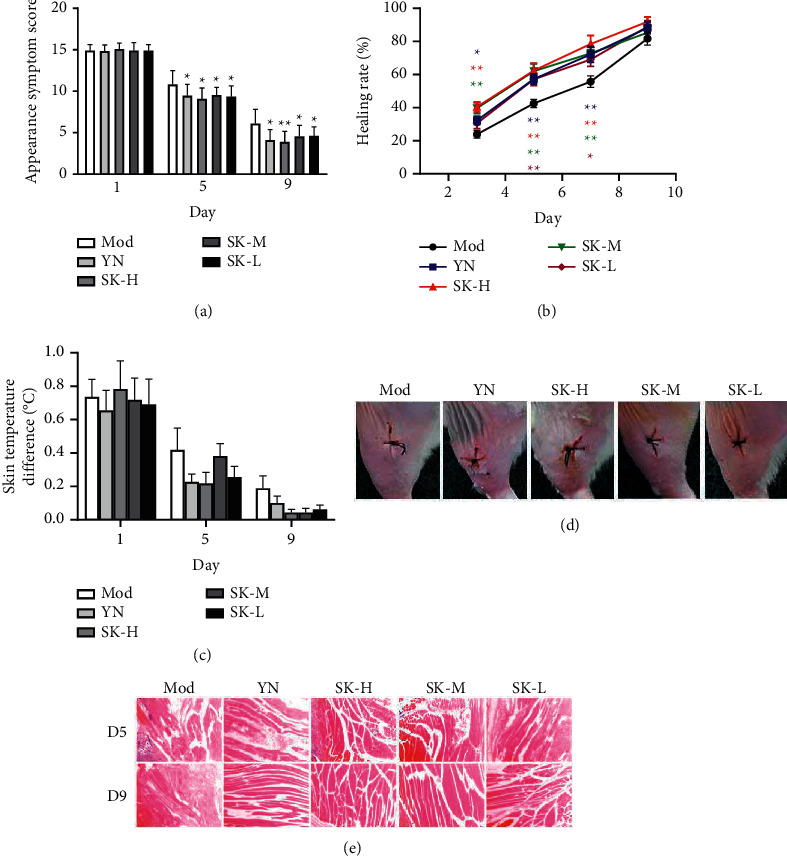
SK attenuated *S. aureus*-induced infectious soft tissue injury in rats. (a) The symptom scores of thigh injury sites. (b) The healing rate of soft tissue cutting site. (c) The temperature difference between the two sides of hind limb. (d) Photos of experimental hind limb. (e) Representative HE staining photos of soft tissue sections from the rats in five different groups (40×). Values are expressed as mean ± SD in each group. ^*∗*^*P* < 0.05 and ^*∗∗*^*P* < 0.01 versus Mod group (*n* = 9).

As shown in Figure 5(d), the symptoms of thigh redness, swelling, and blood stasis could be observed, which indicated the establishment of infectious soft tissue injury model. According to the scoring criteria of soft tissue injury symptoms, compared with the Mod group, the symptom scores of thigh injury sites in each drug group were significantly lower (*P* < 0.05 or *P* < 0.01) on the fifth and ninth day of administration (Figure 5(a)).

The healing rate of soft tissue cutting site in each group was shown in Figure 5(b). On days 3, 5, and 7, compared with the model group, the healing rate of each dosage of SK groups and YN group increased significantly (*P* < 0.05 or *P* < 0.01). On the ninth day, the healing rates of these treatment groups were greater than that in the Mod group, but without statistical difference. Because focal infection of bacterial and soft tissue damage can lead to local skin temperature changes, the skin temperature of rat's hind limb was measured with infrared thermography. The result showed that the temperature difference between the two sides of the hind limb in each treatment group was smaller than that in the Mod group, but without statistical difference (Figure 5(c)).

In addition, the histopathological changes of soft tissues were detected by HE-stained method (Figure 5(e)). On day five, the myofibrils denaturation, a large amount of inflammatory cells infiltration, and broken collagen fibers could be observed in the model group, while the rats of SK-M and SK-H showed relatively less inflammatory cell infiltration; the degree of inflammatory cell infiltration in YN group and SK-L group was slightly higher than that in SK-M and SK-H groups. On day nine, in model group, the infiltration of inflammatory cells was still obvious, the sarcoplasm was swelled due to myofibrillar denaturation, the texture of muscle fiber was unclear, and mucinous degeneration could be seen. However, the drug treatment groups showed slight inflammatory cell infiltration and the striated muscle cells recovered to some extent. A small amount of myofibrillar granule degeneration was observed in SK-L group, and the infected site recovered better in SK-H, SK-M, and YN groups. These results indicated that SK application promoted the recovery of *S. aureus*-induced soft tissue injury.

#### 3.3.2. Effects of SK on Bacterial Contents in the Muscle of Rats

**Figure 6:**
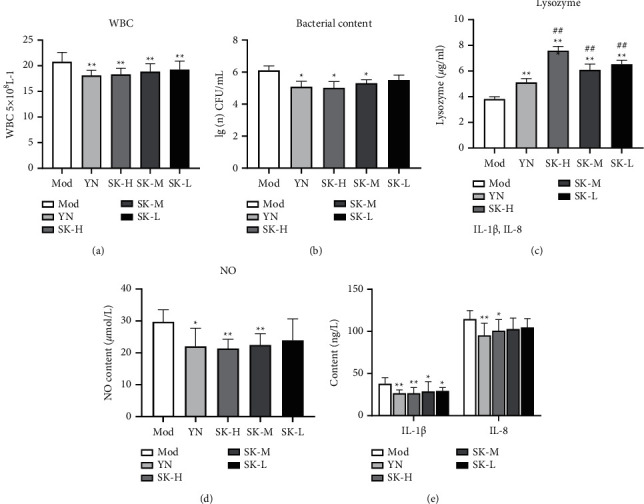
SK treatment ameliorated infectious soft tissue injury in rats. (a) The bacterial content in soft tissue. (b) White blood cells in blood. (c) The contents of lysozyme. (d) NO level in serum. (e) The contents of IL-1*β* and IL-8 in muscles. Values are expressed as mean ± SD in each group. ^*∗*^*P* < 0.05 and ^*∗∗*^*P* < 0.01 vs. Mod group; ^#^*P* < 0.05 and ^##^*P* < 0.01 vs. YN group. (*n* = 8).

The experimental data (Figure 6(a)) showed that the bacterial contents in the muscle of rats with SK or YN treatment were lower than that in Mod group, and there was significant difference between SK-H, SK-M, or YN group and the Mod group (*P* < 0.05).

#### 3.3.3. Effect of SK on Leukocyte Level

Compared with the Mod group, the leukocyte levels of each treatment group were declined significantly (*P* < 0.01), which indicated that SK and YN could inhibit the production of leukocyte to reduce the inflammation of thigh injury site in rats (Figure 6(b)).

#### 3.3.4. Effect of SK on the Contents of Lysozyme

Lysozyme is a kind of hydrolase, also known as cell wall lysozyme, which can destroy the cell wall of *G*^+^ bacteria, but has little effect on *G*^−^ bacteria. As shown in Figure 6(c), compared with the model group, the lysozyme contents in each drug group increased significantly (*P* < 0.01). The contents in SK groups were significantly higher than YN group (*P* < 0.01). The result indicated that SK promoted the secretion of lysozyme to inhibit the growth of bacteria.

#### 3.3.5. Effect of SK on the Levels of NO, IL-1*β,* and IL-8

Inflammatory vascular response and leukocyte response are achieved through the action of a series of chemical factors, such as NO, IL-1*β*, and IL-8. Compared with the model group, NO levels of SK-H, SK-M, and YN groups in serum were obviously decreased (*P* < 0.05) (Figure 6(d)). About the content of IL-1*β* in muscles, all the drug treatment groups were significantly decreased (*P* < 0.05 or *P* < 0.01). Moreover, the content of IL-8 in muscles was significantly lowered in SK-H and YN groups (*P* < 0.05) and that in SK-M and SK-L group decreased but had no obvious difference (Figure 6(e)). The results indicated that SK could obviously inhibit the release of inflammatory factors to reduce inflammation.

#### 3.3.6. SK Inhibited TLR2/MyD88/NF-*κ*B Signaling Pathway

**Figure 7:**
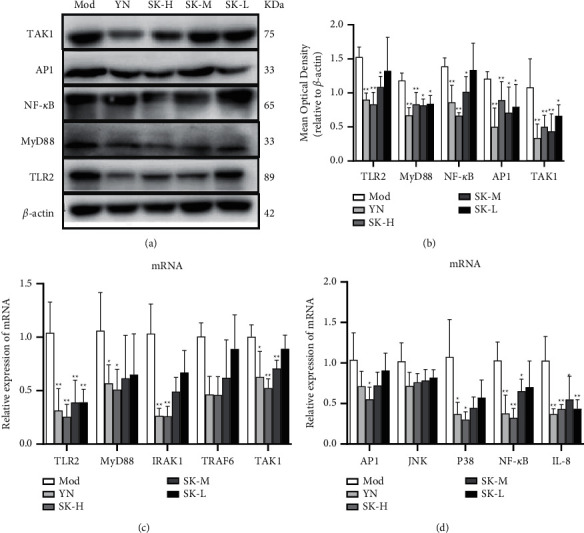
SK inhibited TLR2/MyD88/NF-*κ*B signaling pathway in SSTIs. (a) Representative western blots showed the protein expression of TLR2, MyD88, TAK1, AP1, and NF-*κ*B. (b, c) Effect of SK on the mRNA expression of TLR2, MyD88, IRAK1, TRAF6, TAK1, AP1, JNK, P38, NF-*κ*B, and IL-8 by qPCR. Values are expressed as mean ± SD in each group. ^*∗*^*P* < 0.05 and ^*∗∗*^*P* < 0.01 versus Mod group.

To understand the underlying mechanism on the anti-inflammatory effect of SK, we investigated its effect on TLR2/MyD88/NF-*κ*B signaling pathway. As shown in Figures 7(a) and 7(b), western blot analysis revealed that the expression of TLR2, MyD88, TAK1, AP1, and NF-*κ*B protein in muscle tissues of rats with drug treatment was significantly lower than that in the model group. Compared with the model group, the expression of TLR2 and NF-*κ*B protein in SK-H, SK-M and YN groups decreased significantly (*P* < 0.05 or *P* < 0.01). The expression of MyD88 and AP-1 and TAK1 protein was decreased significantly in all drug treatment groups (*P* < 0.05 or *P* < 0.01).

The results of qRT-PCR (Figures 7(c) and 7(d)) showed that, in response to *S. aureus* infection, the expression of TLR2, MyD88, IRAK1, TAK1, TRAF6, AP-1, p38, NF-*κ*B, and IL-8 mRNA in rats was elevated to high levels, whereas the treatment of SK significantly decreased their expression. Compared with the model group, the mRNA expression of TLR2 and IL-8 in all drug groups decreased significantly (*P* < 0.05 or *P* < 0.01); the expression of IRAK1, TAK1 and NF-*κ*B in SK-H, SK-M, and YN groups decreased significantly (*P* < 0.05 or *P* < 0.01); the expression of MyD88, TRAF6, AP-1, and p38 in SK-H group decreased significantly (*P* < 0.05 or *P* < 0.01). However, the mRNA expression of JNK in drug groups decreased, but there was no statistical difference. It suggested that the anti-inflammatory effect of SK in the treatment of SSTIs may be related to the inhibition of TLR2 signaling pathway.

## 4. Discussion

SSTIs is one of the most common infectious diseases. The most common pathogen is Gram-positive bacteria, which can induce inflammatory cascade by activating Toll-like receptors (TLR) mediated signal transduction pathway, to form inflammatory skin and soft tissue infection [23, 24]. After pathogen-associated molecular patterns (PAMP) activates TLR2, a transmembrane receptor, it interacts with myeloid differentiation factor 88 (MyD88), directly recruits interleukin-1 receptor related protein kinase 1 (IRAK1), interleukin-1 receptor related protein kinase 4, and tumor necrosis factor receptor related factor 6 (TRAF6) to form a complex and phosphorylates IRAK1 to activate its kinase activity. Then, IRAK1 and TRAF are released, and the latter combines with transforming growth factor *β* activated kinase-1 (TAK1) and induces its activation, then activates transcription factors NF-*κ*B and AP1 or activates JNK to act on AP1, and finally produces a large number of biological effector molecules, such as TNF-*α* and IL-8, IL-1*β* [25–28]. Therefore, TLR2/MyD88/NF-*κ*B signaling pathway plays an important role in infectious diseases and inflammatory diseases [29], and inhibiting TLR2 could treat soft tissue infections by inhibiting inflammation and protecting tissues [30, 31].

SK is a well-known compound preparation of traditional Chinese medicine, which had a good clinical effect for many years, and it has been proved to have anti-inflammatory, analgesic, and antibacterial effects. Furthermore, it has less acquired drug resistance, but the therapeutic mechanism is unclear. SK is a herb lotion that mainly consists of a variety of traditional Chinese medicines. Among them, Huanglian, Huangbai, and Zhi-zi have been used for dissipating heat and drying the damp and purging fire for removing toxin. In addition, Zicao has the traditional efficacy of cooling blood and activating blood circulation, detoxification, and rash penetration. Indeed, modern pharmacological studies shows that alkaloids are the main active ingredients of Huanglian and Huangbai, and berberine is the most representative compound of them [32]. It has been reported that berberine has antimicrobial and anti-inflammatory effects *in vivo* and *in vitro* [33–35]. Geniposide is a key bioactive component of Zhi-zi. It is one of the iridoid compound extracted from Zhi-zi and has been shown to have anti-inflammatory and other pharmacological effects [36, 37]. It has been found that shikonin is one of the main active ingredients of Zicao, with anticancer, anti-inflammatory, and wound-healing effects [38, 39].

In this study, we established three animal models of SSTIs by *S. aureus*, including skin layer, subcutaneous tissue, and muscle infections, and we selected Mupirocin ointment (a kind of topical antibiotics) as the positive drug in the subcutaneous abscess and dermal ulcer models [40] and Yunnan Baiyao (a kind of compound preparation) as the positive drug in the soft tissue infection model [41, 42]. In the previously mentioned three models, treatment with SK resulted in smaller lesions and reduced the bacteria contents and the infiltration of inflammatory cells compared with the model control groups, which indicated that SK has anti-inflammatory and antibacterial effects on SSTIs. In the dermal ulcer rat model, SK inhibited TAK1/NF-*κ*B and TAK1/p38 signaling pathway and also inhibited the expression of TNF-*α*, an inflammatory factor involved in whole inflammatory process [43]. Furthermore, through detecting the healing rate of skin ulcer and the hydroxyproline contents in local tissue, we found that SK can not only control local infection symptoms but also promote ulcer healing.

Therefore, in the model of *S. aureus-*induced infectious soft tissue injury, we studied TLR signaling pathway, the upstream regulators of TAK1. The results showed that SK quickly controlled the infection of muscle tissue and promoted the recovery of soft tissue injury. TLR2/NF-*κ*B pathway plays an important role in bacterial infectious diseases. The downregulation effect of TLR2/MyD88/NF-*κ*B expression indicated that SK cured the soft tissue infections because it effectively prevented the development of inflammatory response induced by the TLR2 ligands of *S. aureus*. Furthermore, SK inhibited TAK1, NF-*κ*B, p38, and AP1 but had no significant effect on JNK, which suggested that SK mainly inhibited the downstream signals as p38 and NF-*κ*B.

## 5. Conclusion

In conclusion, this present study first demonstrated that SK had obvious anti-inflammatory and antibacterial effects on the *S. aureus* infected SSTIs, such as subcutaneous abscess, dermal ulcer, and infectious soft tissue injury models, which provide an effective therapeutic method for SSTIs. The involved mechanism may be related to the inhibition of TLR2/MyD88/NF-*κ*B signaling pathway related factors expression and then the reduction of the inflammatory response. Elucidating the advanced mechanism of action requires further study.

## Data Availability

The data used in this study are available from the corresponding author on reasonable request.
